# Towards Ligand Docking Including Explicit Interface Water Molecules

**DOI:** 10.1371/journal.pone.0067536

**Published:** 2013-06-28

**Authors:** Gordon Lemmon, Jens Meiler

**Affiliations:** 1 Center for Structural Biology, Vanderbilt University, Nashville, Tennessee, United States of America; 2 Department of Chemistry, Vanderbilt University, Nashville, Tennessee, United States of America; Weizmann Institute of Science, Israel

## Abstract

Small molecule docking predicts the interaction of a small molecule ligand with a protein at atomic-detail accuracy including position and conformation the ligand but also conformational changes of the protein upon ligand binding. While successful in the majority of cases, docking algorithms including RosettaLigand fail in some cases to predict the correct protein/ligand complex structure. In this study we show that simultaneous docking of explicit interface water molecules greatly improves Rosetta’s ability to distinguish correct from incorrect ligand poses. This result holds true for both protein-centric water docking wherein waters are located relative to the protein binding site and ligand-centric water docking wherein waters move with the ligand during docking. Protein-centric docking is used to model 99 HIV-1 protease/protease inhibitor structures. We find protease inhibitor placement improving at a ratio of 9∶1 when one critical interface water molecule is included in the docking simulation. Ligand-centric docking is applied to 341 structures from the CSAR benchmark of diverse protein/ligand complexes [1]. Across this diverse dataset we see up to 56% recovery of failed docking studies, when waters are included in the docking simulation.

## Introduction

Small molecule docking methods predict the structure of a protein/ligand complex [2]. Ligand docking consists of two components: s*ampling* of the conformational space, and *scoring* of the resultant structures [3]. Sampling of the conformational space typically includes ligand position with respect to the protein often called ‘pose’), ligand conformation, and protein conformation. Scoring seeks to distinguish correct from incorrect binding poses by estimating binding affinity. It is characterized by a trade-off between accuracy and speed [3], [4]. While current sampling and scoring algorithms are often able to predict the correct binding pose [5], satisfactory prediction of binding affinity has yet to be achieved [4]. One particular challenge in ligand docking studies is the positioning of interface water molecules [5].

That interface water molecules play an important role in ligand binding is evidenced by the fact that many protein/ligand complexes contain structured waters that bridge protein and ligand. For instance in the CSAR dataset 299 out of 341 complexes include waters within hydrogen bonding distance of both protein and ligand atoms. These water molecules are often absent in experimental structures of the apo protein [6]. Waters stabilize protein/ligand interfaces by providing indirect interactions between protein and ligand through formation of hydrogen bonds with both partners [7]. In empirically derived scoring functions optimized to predict binding affinities [8], [9], components such as hydrogen bond energy have been weighted to account for the change in energy compared to hydrogen bonds formed with water [10]. Similarly the “hydrophobic” score terms are used to represent desolvation of the protein receptor. Nevertheless, great improvements have been seen in molecular dynamics based binding affinity prediction when water is considered [11], [12].

For the present study we introduce the notions of “protein-centric” and “ligand-centric” water docking. Protein-centric waters move independent of the ligand. In the ligand-centric approach waters placed around the ligand move with the ligand during initial ligand placement, and then move independently. The protein-centric approach has the advantage that often likely water positions are known from crystallographic studies. An advantage of a ligand-centric approach is that since the surface of drug-like ligands is typically smaller than the protein binding interface, fewer water positions need to be considered. So far, mostly protein-centric approaches have been tested.

In both self-docking [13] and cross docking studies [14], correct ligand binding pose prediction can be improved by the presence of conserved crystallographic waters. For instance a FlexX prediction of an HIV-1 protease/protease inhibitor interface fails without the inclusion of a key water, But prepositioning this water at its known crystallographic coordinate leads to a practically perfect prediction [15]. In this case the effect of water had little to do with *scoring* and everything to do with guiding the *sampling* algorithm. De Graaf et al. find RMSD accuracy improved 18% for AutoDock, 23% for FlexX, and 11% for GOLD when crystallographic waters were included [16] in Cytochrome P450 binding sites. Inclusion of crystallographic waters in the thymidine kinase binding site leads to 17% (Autodock), 35% (FlexX) and 0% (GOLD) improvements in RMSD prediction.

Nevertheless, explicit prediction of the *location* of key water molecules when docking ligands is not standard in current docking algorithms and limited to few specific examples: In a protein-centric approach, De Graaf et al. used GRID to preposition potential water positions within the binding pockets of 19 cytochrome P450 and 19 thymidine kinase crystal structures. These waters were present during docking predictions using AutoDock, FlexX, and GOLD. The authors found RMSD accuracy improved by 70% (Autodock), 32% (FlexX) and 7% (GOLD) for Cytochrome P450 docking 23% (Autodock), 12% (FlexX) and 23% (Gold) in RMSD placement for thymidine kinase [16].

Lie et al. present a *ligand-centric* model for docking with waters. Waters are placed around and move with the ligand. The authors chose 12 protein/ligand complexes in which docking studies without water failed and docking studies that consider all crystallographic water molecules succeed. Results from docking with ligand-centric waters demonstrate top ranked models with RMSD less than 2.0 Å in 6 out of 12 cases [17]. Note that this study will not notice if addition of waters leads to failures in cases that were successful without addition of waters.

RosettaLigand [18] has proven effective at generating models of protein/ligand complexes at atomic-detail accuracy (<2.0 Å) [19], [20]. RosettaLigand samples protein and ligand flexibility simultaneously [20]. Recent updates to RosettaLigand software have allowed for docking multiple small molecules (including waters, metals, and cofactors) simultaneously [21]. We use this new feature to push the boundary of ligand docking with water molecules in several ways: (1) the water is not held fixed with respect to protein or ligand position. (2) RosettaLigand allows both *protein-centric* and *ligand-centric* water placement. (3) Protein flexibility and ligand flexibility are considered. (4) We use a much larger dataset of 341 diverse protein/ligand complexes [1] to provide a more stringent and comprehensive benchmark than previous studies. We benchmark Rosetta using (1) a set of HIV-1 protease/inhibitor co-crystal structures from the protein data bank (PDB, www.rcsb.org), and (2) the CSAR benchmark dataset [1] (www.csardock.org).

HIV-1 protease (PR) plays an essential role in the HIV-1 lifecycle and thus is an important target for drug therapy [22]. PR is the classic success story of structure-assisted drug design [23]. The binding of most HIV-1 protease inhibitors (PIs) is mediated by a key water molecule that forms hydrogen bonds between the PI and the PR’s flexible loop regions [24]. This interaction is necessary for binding and stabilizes the loops in the closed-conformation [25]. We selected 11 protease/protease inhibitor complexes from the PDB. These include 9 different protease inhibitors and a variety of different protease sequences (due to mutation). We perform cross-docking studies between pairs of protease structures by combining inhibitor and sequence from one PR/PI complex with backbone coordinates from another. We do so using standard docking as well as docking with protein-centric waters with positions identified through crystallographic studies. Our results demonstrate significant improvement in binding pose prediction when water docking is included.

In addition to a homogeneous and well-understood benchmark, we assess the effect of water docking on a benchmark of heterogeneous protein/ligand complexes. The CSAR benchmark includes 341 protein/ligand complexes experimentally determined structures of protein/ligand complexes. Each CSAR datapoint also contains structural waters and K_d_ values. CSAR data was prepared for the uniform evaluation of methods for prediction of ligand binding mode and binding affinity. In 195 of these structures, we find between 1 and 8 water molecules positioned to directly interact with both protein and ligand. Unlike the HIV-1 PR dataset, wherein extensive structural and biochemical studies have confirmed the importance of the key water molecule studied, the waters we study within the CSAR dataset were chosen simply based on their crystallographic coordinates. In this paper the CSAR dataset is subjected to both standard docking without water molecules and docking with ligand-centric waters. We find significant improvement in model ranking when waters are added. Inhibitor RMSDs improve slightly.

## Materials and Methods

### Preparation of HIV-1 PR Inputs for Cross Docking

**Figure 1:**
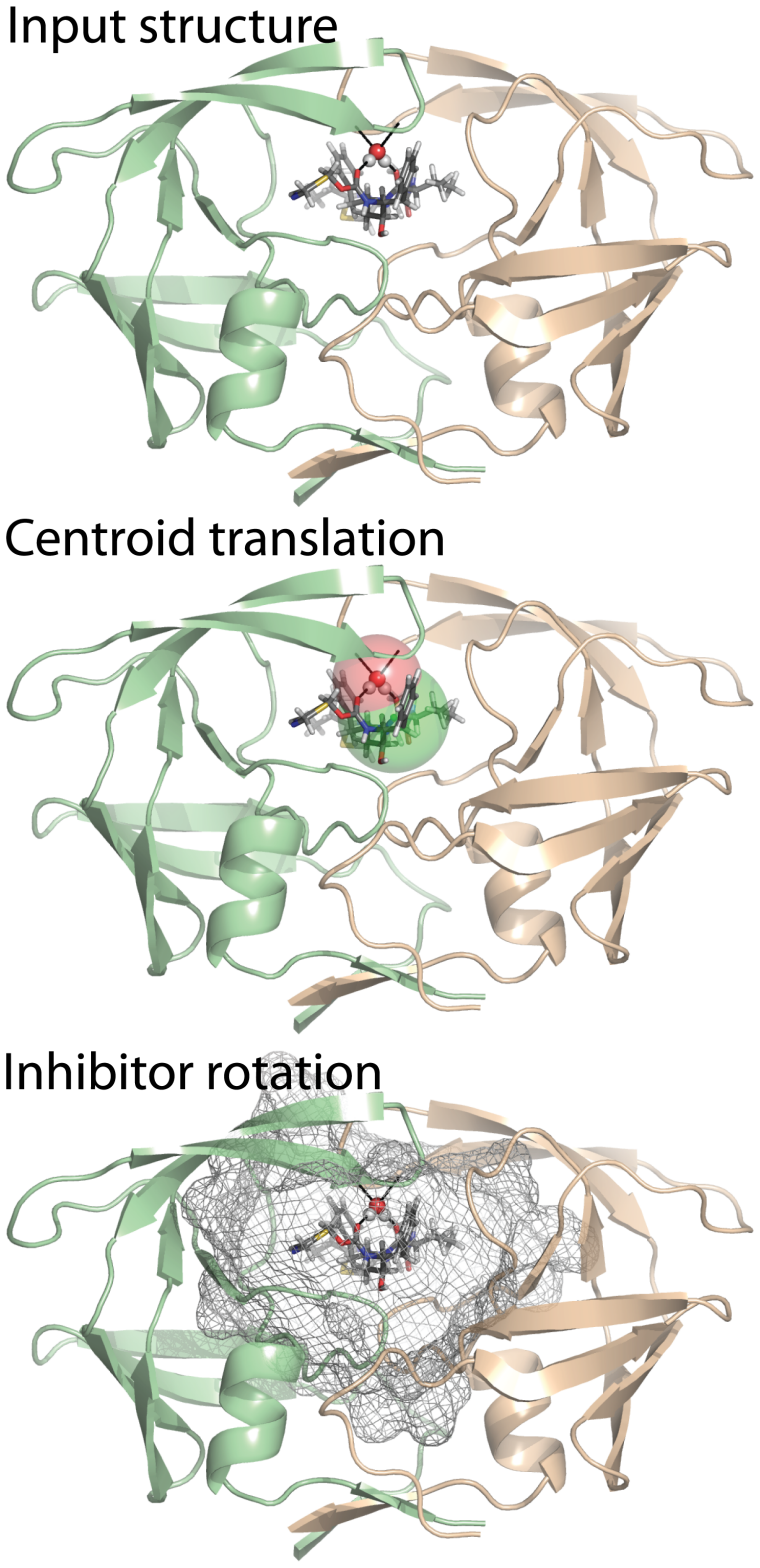
Sampling of the HIV-1 protease binding pocket by ritonavir and a conserved water. Top: One key water molecule hydrogen bonds (black lines) with both HIV-1 PR flexible flaps and protease inhibitor Ritonavir. Middle: Standard docking begins with translation of the inhibitor from its centroid, by up to 5 Å (green sphere). Protein centric water docking also includes up to 4 Å translation of water (red sphere). Bottom: Grey mesh indicates sampling space covered after ligand rotation. Image was prepared using Pymol. The structure shown was downloaded from the protein databank (PDB ID: 1HXW).

Eleven HIV-1 PR crystal structures representing 9 unique PR sequences bound to one of 11 unique protease inhibitors (PIs) were obtained from the PDB. Each of these structures includes a conserved water molecule, known to be important for stabilizing loop regions during binding (Figure 1, top panel). The PR sequences represented herein differ from one another by up to 14 residues per 99 residue chain. RMSDs between each pair of HIV-1 PR crystal structures are shown in Table S2 in File S1. Cross docking consists of combining the PI and PR sequence from one complex with the backbone coordinates of another complex. Each PI was combined with each protease backbone, producing 99 input structures. Appropriate side chain coordinates were added in the docking simulation. Rosetta ligand docking is challenged to correctly predict PI pose, given the imperfect backbone starting coordinates. In our study each of the 99 input structures is docked with and without the inclusion/docking of the conserved water molecule mentioned above.

### Preparation of CSAR Dataset

First we extracted ligand atom coordinates from the input files. Rosetta software ships with a script, `mol_file_to_params.py` which was used to prepare.params files describing the chemical properties of each ligand and assigning each ligand atom a Rosetta atom type. We wrote scripts that use BioPython to right align residue names, convert non-canonical residues to their canonical base residues, and remove neutralizing caps from N-terminal and C-terminal ends of CSAR input structures. Protein chains were relabeled alphabetically, as they appear in the PDB. The ligand was given the chain ‘X’ and residue code ‘INH’. All waters were given the chain ID ‘W’ and the residue code ‘WAT’. We wrote a python script that uses PyMOL [26] to select interface waters from among all waters in the crystal structure. Access to this script is described in Protocol S4 in File S1. ‘Loose waters’ were defined as those with oxygen atoms within 3.0 Å of at least one protein and one ligand atom. ‘Tight waters’ have oxygen atoms within 3.0 Å of at least 2 protein and two ligand atoms. Finally for each ligand we used the Biochemical Library (BCL), to determine LogP, molecular weight, number of rotatable bonds, number of hydrogen bond acceptors, and number of hydrogen bond donors. BCL is a cheminformatics software package freely downloadable from www.meilerlab.org.

### Standard Docking – low Resolution Sampling

For both HIV-1 PR and CSAR data, docking without waters entails, first placing the ligand in the putative binding site. Next the ligand is translated randomly within a 5 Å radius sphere (Figure 1, middle panel, green sphere). This is repeated up to 50 times, or until the ligand centroid does not clash with the protein in the new ligand position. If after 50 cycles of movements no non-clashing placement is identified, the placement with the lowest score is accepted. Next comes a rotation step in which the ligand is rotated randomly up to 1000 times to identify a rotation that does not lead to clashes with the protein. Unlike the translation step, which only considers the ligand centroid, the rotation step affirms that no ligand atoms clash with protein atoms. The “slide together” step then slides the ligand toward the protein until they touch. This step ensures the ligand is in close enough proximity to the protein to allow the high affinity contacts to be formed during high resolution docking. The space sampled by this low resolution protocol is represented as mesh, Figure 1, bottom panel.

### Standard Docking – high Resolution Refinement

High resolution docking involves small ligand translations of up to 0.1 Å and rotations of up to 5°. These movements are coupled with either rotamer trials (sampling of rotamers, one residue at a time) or repacking (sampling rotamers at multiple positions simultaneously). Both rotamer trials and repacking are restricted to residues within 6 Å of any ligand atom. Next a gradient based minimization is applied, which allows for interface side-chain torsion angle adjustments, along with adjustment of ligand torsion angles. High resolution docking is repeated 6 times, using a Monte Carlo approach. During a final minimization step, backbone φ/φ and side chain χ angles within 7 Å of the ligand as well as all ligand torsion angles are minimized. This high resolution refinement was included in both HIV-1 PR and CSAR docking. The Rosetta XML defining this standard docking protocol is included as Protocol S1 in File S1.

### HIV-1 Protease/Protease Inhibitor Cross-docking with a Protein-centric Water

The 99 HIV-1 PR/PI cross-docking inputs were subjected to a docking protocol in which one key water moves independent of the ligand. In this protein-centric water docking scheme, the interface water is initialized at its crystallographic coordinates. During translation, this water is allowed to move within a sphere with a 4 Å radius (Figure 1, middle panel, red sphere). During rotation, the water is allowed to fully reorient. As hydrogen is generally not resolved in X-ray crystal structures, Rosetta adds hydrogen to the water molecule prior to translation or rotation. The interface definition used to select residues for side-chain repacking and for backbone minimization was extended to include the residues close to the water molecule. During high resolution docking, this water is allowed to move in the same fashion as the ligand –0.1 Å translations and 5° rotations. Table 1 compares protein-centric water docking with other protocols used in this study. The Rosetta XML defining protein-centric docking is provided as Protocol S2 in File S1.

**Table 1:** Comparison of protocols for the 2 benchmark studies presented in this paper.

	Protein Centric Water docking (HIV-1 PR/PIs)	Ligand Centric Waters (CSAR benchmark)
Input preparation	Crystallographic waters within 3.0 Å of protein and ligand are included in the docking study	
Ligand Translation	**Ligand** moves up to 5 Å, finding a non-clashing location.	**Ligand & water** move together up to 5 Å, finding a non-clashing location.
Water Translation	Up to 50 cycles of 1 Å water movement, first non-clashing move is accepted.	
Ligand Rotation	**Ligand** rotates up to 1000 times to optimize attractive & repulsive scores	**Ligand & water** rotate together up to 1000 times to optimize attractive & repulsive scores
Water Rotation	Waters rotate together up to 100 times to optimize attractive & repulsive scores	
High Resolution docking	6 Cycles of ligand & water translation (0.1 Å) and rotation (5°). Each cycle coupled with side-chain rotamer sampling & gradient based minimization of side-chain and ligand torsion angles.	
Final minimization	Gradient based minimization of backbone and side chain degrees of freedom around the ligand and waters.	

### CSAR Self-docking with Ligand-centric Waters

The 341 CSAR inputs (described above) were subjected to a docking protocol in which waters are translated and rotated along with the ligand, before they are allowed smaller independent movements. First waters move with the ligand up to 5 Å. Next waters are allowed additional movements of up to 1 Å. The ligand and waters are then allowed full rotation as a single rigid body. Finally waters rotate independently. High resolution docking occurs as described for *protein-centric* waters. Table 1 describes the differences between ligand-centric and protein-centric waters. The Rosetta XML defining ligand-centric docking is provided as Protocol S3 in File S1.

### Placement of Decoy Waters

As part of a control study we place ‘decoy’ waters at reasonable hydrogen bonding distances from every CSAR ligand hydrogen bond donor and acceptor atom. Each water was placed using the following scheme. An XYZ-grid was created with gridpoints spaced 0.15 Å apart. The ligand coordinates were translated to the grid origin. Around each ligand atom was drawn a sphere with a radius equal to its van der Waals radius. Grid points within these spheres were marked as occupied. Next around each ligand hydrogen bond donor or acceptor was drawn a ring with an inner radius of 2.75 Å and an outer radius of 2.9 Å. Grid points that fell within this shell were filtered to remove grid points occupied by other atoms. Finally, for each set of grid points, the central grid point with the shortest average distance to all remaining grid points in the set was chosen as the coordinate for water placement.

### Docking Model Production and Analysis

For both HIV-1 protease and the CSAR dataset a similar approach was used. Regardless of whether we used standard docking, *protein-centric* water docking, or *ligand-centric* water docking, 1000 models were produced per input complex. The top 100 by total Rosetta energy score were selected from among these models. From this subset, RMSDs of top models by ligand interface score are reported throughout this manuscript. Computation was split between the Vanderbilt University ACCRE cluster (www.accre.vanderbilt.edu) and the Center for Structural Biology piranha cluster (structbio.vanderbilt.edu/comp/hw/piranha). Rosetta revision 49194 was used for all calculations.

### Ranking Metrics

Sets of 100 top scoring models described in the previous paragraph were sorted by interface score. Their order of appearance in this sorted list represents their rank. Ranking metrics used in this paper include (1) whether the top ranked model has an RMSD under 2.0 Å, (2) whether there exists a model under 2.0 Å RMSD within the top 10 ranked structures, (3) the change in rank between top scoring models from two separate studies.

### RMSD Calculations

The accuracy of models created by RosettaLigand docking was determined by comparing them to the experimentally determined structures, via root-mean-square deviation (RMSD) calculations. We calculate RMSD by (1) summing over the squared distance for each pair of matching ligand non-hydrogen atoms between experimental and predicted structures, (2) dividing by the total number of ligand atoms, and (3) determining the square root. In the case of cross-docking studies (HIV-1 PR dataset) all input structures were aligned prior to docking. Because the ligand docking protocol includes backbone flexibility only for residues within the interface, the global coordinate system is preserved and it is not necessary to realign experimental structures and Rosetta models before calculating RMSD.

## Results and Discussion

### Protein-centric Docking of a Key Water Molecule Improves Placement of HIV-1 Protease Inhibitors

**Figure 2:**
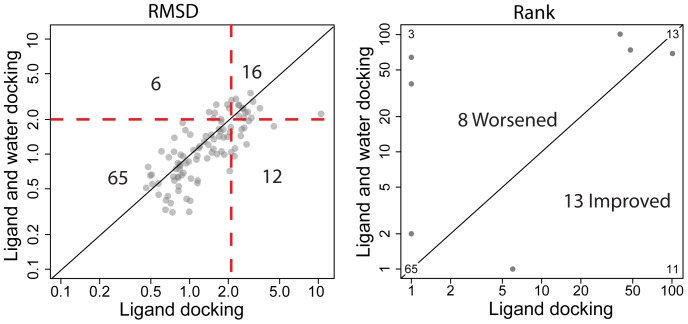
RMSD and rank comparisons between standard and protein-centric water docking of HIV-1 PR/PI. Left panel: RMSD of top scoring Rosetta model. 69 models fall below the diagonal (improved RMSDs) while 30 lie above it. Red dashed lines represent the 2 Å RMSD metric for successful docking. Predictions in the lower-right quadrant turn from failures to successes up on water docking. Upper-left quadrant contains predictions that succeeded without water docking and fail with water docking. Right panel: rank of the lowest scoring Rosetta model with RMSD under 2 Å. Where multiple HIV-1 cross-docking predictions achieved the same rank with and without water docking, these points are replaced with text indicating the number of overlapping points.

The 99 cross-docking PR/PI input structures were subjected to standard docking (without water) and protein-centric water docking, which involves sampling the position and orientation of the conserved water within a 4 Å sphere centered at the crystallographic coordinates (see methods). In 69 out of 99 cross docking studies, the addition of water led to top scoring models where the inhibitor was placed more accurately (Figure 2, left panel). When focusing on only significant changes in RMSD larger than 1 Å in magnitude, we observe 9∶1 ratio of improved to worsened cases (Table S1 in File S1). Note that the 11 cells in Table S1 with matching row and column PDB-IDs represent self-docking results rather than cross docking. Another metric for successful docking is whether the top scoring model has a ligand placed within 2.0 Å RMSD from the experimentally determined position. By this metric twelve failed studies became successes upon addition of water. Yet 6 successful standard docking studies failed when water was added (Figure 2, left panel). Thus RosettaLigand *protein-centric* docking is twice as likely to improve docking results in this particular benchmark.

### Protein-centric Docking of a Key Water Improves Ranking of HIV-1 Protease Inhibitors

One metric used to gauge success in docking is the rank of the first model with RMSD under 2.0 Å. That is, in a list of models sorted by Rosetta predicted interface energy, what is the position of the first model in that list with an inhibitor less than 2.0 Å RMSD from the native coordinates. By this metric 13 ranks improve and 8 get worse when water docking is included in modeling of the PR/PI interface (Figure 2, right panel). In this study 1000 models were produced for each of the 99 PR/PI inputs. It is important to note that increased sampling is likely to improve results within both standard and protein-centric water docking. Our results therefore demonstrate that sampling is improved on average when a key water is docked along with the protease inhibitor.

### Number of CSAR Interface Waters Scales with Ligand Size

The CSAR dataset contains 341 protein/small molecule complex crystal structures [1], each with a reported binding affinity (K_d_). The proteins range in size from 119 residues to 2228 residues. The ligands range in size from 9 atoms to 118 atoms. Other properties are summarized in Table 2. We filtered crystallographic water molecules based on two criteria: ‘loose waters’ are within 3.0 Å of both a protein and a ligand atom; ‘tight waters’ are within 3.0 Å of at least 2 protein and 2 ligand atoms. The tight water subset includes an average of 1.1 waters per complex, while the loose water subset retains 3.3 waters per complex on average. Figures S1 and S2 in File S1 reveal how various ligand properties trend with number of interface waters within the loose and tight subsets, respectively. As expected, the size of the small molecule (as measured by molecular mass, number of rotatable bonds, or number of hydrogen bond donors or acceptors) correlates with the number of water molecules that form interactions with the ligand and the protein.

**Table 2:** Summary statistics describing the CSAR dataset.

	Min	Max	Mean	Median
# of protein residues	119	2228	495	366
# of protein atoms	1756	32736	7664	5661
# of ligand atoms	9	118	42	37
ligand molecular weight	59.1	779	332	304
# of ligand rotatable bonds	0	27	6	5
# of ligand H-bond acceptors	1	24	7	6
# of ligand H-bond donors	0	14	3	3
LogP	−44.9	9.2	−4.6	−1.0
Tight waters	0	8	1	1
Loose waters	0	19	3	3

### Ligand-centric Docking of Interface Waters Improves Placement of CSAR Ligands

**Figure 3:**
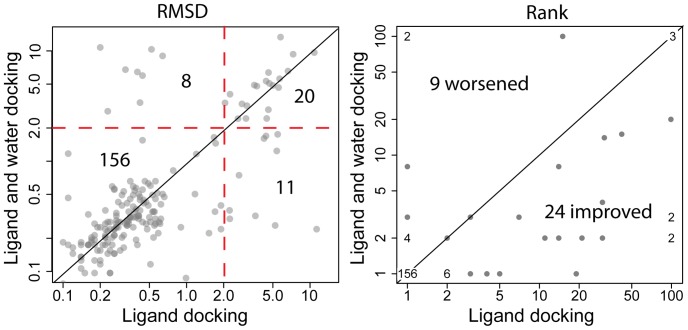
RMSD and rank comparisons between standard and ligand-centric *tight* water docking of CSAR dataset. Left panel: RMSD of top scoring Rosetta model. 106 models fall below the diagonal (improved RMSDs) while 82 lie above it. Red dashed lines represent the 2 Å RMSD metric for successful docking. Predictions in the lower-right quadrant turn from failures to successes up on water docking. Upper-left quadrant contains predictions that succeeded without water docking and fail with water docking. Right panel: rank of the lowest scoring Rosetta model with RMSD under 2 Å. Where multiple CSAR docking predictions achieved the same rank with and without water docking, these points are replaced with text indicating the number of overlapping points.

**Figure 4:**
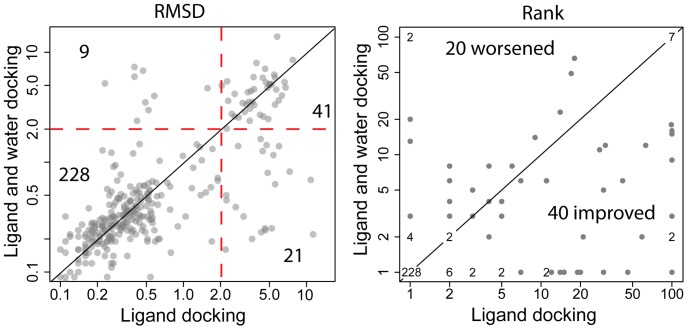
RMSD and rank comparisons between standard and ligand-centric *loose* water docking of HIV-1 PR/PI. Left panel: RMSD of top scoring Rosetta model. 159 models fall below the diagonal (improved RMSDs) while 129 are above it. Red dashed lines represent the 2 Å RMSD metric for successful docking. Predictions in the lower-right quadrant turn from failures to successes up on water docking. Upper-left quadrant contains predictions that succeeded without water docking and fail with water docking. Right panel: rank of the lowest scoring Rosetta model with RMSD under 2 Å. Where multiple CSAR docking predictions achieved the same rank with and without water docking, these points are replaced with text indicating the number of overlapping points.

As described in the methods, crystallographic waters in proximity to interact with the ligand were included in ligand-centric docking of CSAR data. These waters initially moved with the ligand and are subsequently allowed to translate and rotate independent of the ligand. Table 3 shows average scores and RMSD of top scoring Rosetta models with and without water docking. With both tight and loose water subsets, Rosetta energy scores decrease when water is docked. No significant change is seen in average ligand placement accuracy (RMSD, Table 3) when water is also docked. However, counts of the number of improved RMSDs and worsened RMSDs demonstrate that ligand-centric water docking is more likely to improve ligand placement than to make it worse. Ratios of improved to worsened RMSDs for the tight and loose subsets are 106∶82 and 159∶129 respectively (see left panels of Figures 3 & 4).

**Table 3:** Mean values for top models from Rosetta CSAR docking results.

Waters	Protocol	N	Total score	Ligand	RMSD	Water	W_RMSD
Tight	Standard dock	195	−1192±954	−17.93±6.5	1.06±1.79		
	Water dock	195	−1197±953	−20.80±7.4	1.18±2.26	−3.56±2.29	1.48±1.48
	Water – Standard	195	−4.6±14.8	−2.87±2.39	0.12±1.97		
	Per water effect	195	−1.67±8.13	−1.61±1.28	−0.01±1.47	−2.49±2.48	0.98±1.12
Loose	Standard Dock	299	−1184±968	−17.28±6.3	1.24±1.86		
	Water dock	299	−1193±968	−21.11±7.6	1.09±1.80	−3.20±1.86	1.60±1.38
	Water – Standard	299	−8.8±16.4	−3.83±3.36	−0.15±1.69		
	Per water effect	299	−1.86±5.71	−1.04±0.92	−0.04±0.78	−1.32±1.29	0.65±0.84

‘Ligand’ is the component of total energy contributed by the presence of the ligand. ‘RMSD’ is calculated by comparing experimental and predicted ligand coordinates. ‘Water’ is the component of total energy contributed by the presence of waters. ‘W_RMSD’ is calculated by comparing experimental and predicted water coordinates. Rows 3 and 7 represent the difference between standard docking and ligand-centric water docking. ‘Per water effect’ reports the mean score and RMSD values after dividing individual values by the number of waters present in the study.

### Ligand-centric Docking of Interface Waters Improves Ranking of CSAR Ligands

**Figure 5:**
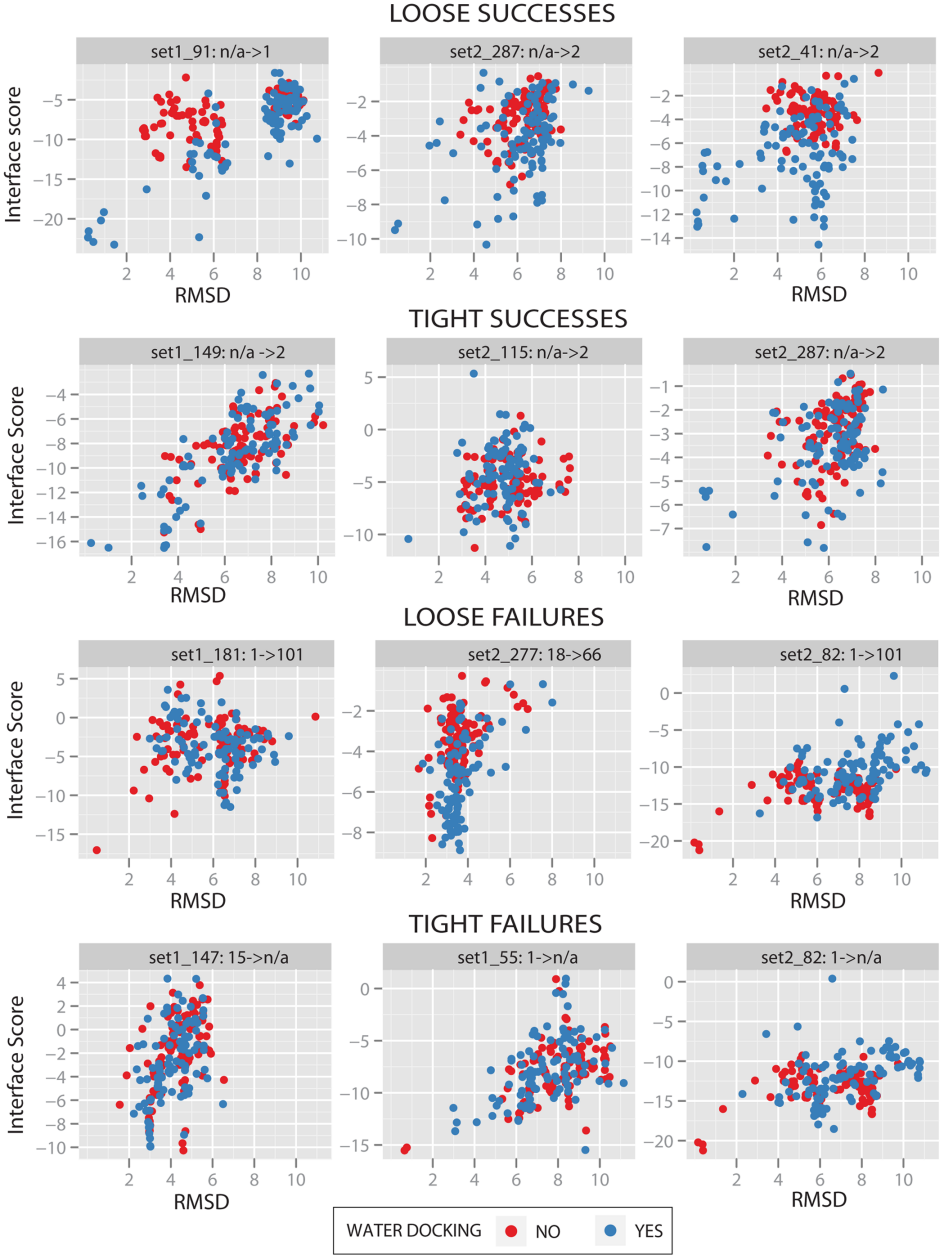
RMSD vs Rosetta interface score for CSAR predictions. Each plot contains the top 100 Rosetta models by total score for both standard (red) and water (blue) docking for particular CSAR datapoint. Each plot is identified by its CSAR label (e.g ‘set1_91’). CSAR labels are followed by rank before and after water docking. Ranks of ‘n/a’ indicate that no model below 2 Å RMSD was sampled by Rosetta. Each set of 3 plots represent the largest rank changes seen in that category. Successes are defined as ranks that decrease and failures as ranks that increase.

The rank of the first Rosetta model (by interface score) under 2 Å RMSD is a common measure of prediction quality. Figure 3 (right panel) demonstrates that ligand-centric docking of tight waters improves ranks 24 times for every 9 times it makes them worse. Similarly, loose water docking improves ranks twice as often as it worsens them (Figure 4, right panel). Figure 5 graphs score vs. RMSD with and without water docking for several CSAR models. These plots reveal that the effect of water docking is related to improved sampling rather than improved scoring. This is evidenced by the presence of water-docking models below 2 Å RMSD where standard docking failed to sample models below 2 Å RMSD. If water docking produced benefits through improved scoring, then below 2 Å RMSD models would be present in standard docking results, but have been given unfavorable scores.

### Differences between CSAR Standard Docking and CSAR Ligand-centric Water Docking are Statistically Significant

**Figure 6:**
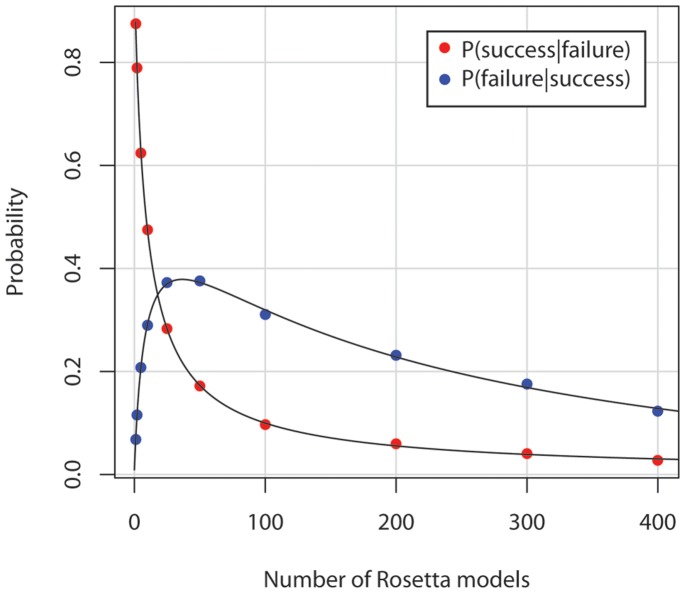
Probability of changes in CSAR docking success upon replication of docking study. Success is measured as whether the top scoring model by interface score has a ligand pose within 2.0 Å RMSD of the native pose. As sampling size increases, the probability that resampling with would change the outcome of docking decreases. Equations for the best-fit lines are available in Table S4 in File S1.

We calculate the probability that, for a given a sampling size, resampling would lead to a change in whether docking was ‘successful’ (Table S3 in File S1). Success is measured as whether the top scoring structure by interface score has an RMSD under 2.0 Å. Using Bayes theorem we calculate conditional probabilities of a success becoming a failure and a failure becoming a success (Table S3 in File S1). These conditional probabilities are plotted in Figure 6 as a function of model number. Equations that best fit this data suggest that the probability of a 1000 model CSAR docking study failing upon replication of the study is around 2.3% whereas the probability of a failed docking study succeeding upon replication is around 8.8% (see Table S4 in File S1). We use these probabilities to test the null hypothesis using a one-tailed binomial distribution. All p-values are well below 0.05 (Table 4, columns 2–3). Since we cannot guarantee the validity of our extrapolations, we also calculate p-values using observed probabilities for N = 400 found in Table S3 in File S1. These p-values (Table 4, columns 4–5) are also well below 0.05. Thus, the improvement in the numbers of successful docking studies is extremely unlikely to have resulted from random chance.

**Table 4:** P-values calculated using a one-tailed binomial distribution.

	N = 1000		N = 400	
	Tight	Loose	Tight	Loose
Number of standard dock successes	164	237	164	237
Probability resampling leads to failure	0.0136	0.0136	0.028	0.028
# of successes that become failures upon ligand-centric water docking (K)	9	20	9	20
P(K or more)	4.6e-4	1.57e-10	0.042	1.47e-5
Number standard dock failures	31	62	31	62
Probability resampling leads to success	0.0878	0.0878	0.123	0.123
# of failures that become successes upon ligand-centric water docking (K)	24	40	24	40
P(K or more)	<1e-12	<1e-12	<1e-12	<1e-12

Probabilities in columns 2–3 are extrapolated using best-fit lines from Figure 6 assuming 1000 models are produced. Probabilities in columns 4–5 were observed for N = 400 (from Table S3 in File S1, last row). Whether p-values are calculated using extrapolated probabilities or probabilities observed for N = 400, it is clear that changes in docking success cannot be attributed to chance.

### Random Water Placements do not Lead to Improved Docking Results

We sought to perform a control study to demonstrate that our improved *ligand-centric* docking results were not due to the addition of water generally, but due specifically to the correct placement of waters relative to ligands as they appear in experimentally determined structures. To accomplish this we filtered the CSAR dataset to obtain 45 complexes with only 1 interface water. For each of the 45 ligands from these complexes waters were placed at distances that would allow hydrogen bonding between ligand donor and acceptor atoms (see Methods). During the rotation step of ligand-centric low-resolution ligand placement, one water molecule that does not clash with the protein is chosen at random from among these putative water placements.

Ligand-centric high resolution docking proceeds with independent docking of the small molecule and the selected decoy water. We generate 1000 models per 45 input structures. We rank these results as described previously and find that ligand-centric docking with decoy waters lead to 7 improved ranks and 10 worsened ranks as compared to standard docking results. In contrast ligand-centric docking with crystallographic water positions leads to 10 improvements and only 2 worsened results. We conclude that waters placed near randomly selected ligand polar groups increase the conformational search space drastically leading to a reduced probability for Rosetta to identify the correct pose in 1000 docking trials. With a more exhaustive sampling approach Rosetta could be used to predict the positions of both the ligand and interface waters.

### Spacious Binding Pockets are more Likely to Benefit from Ligand-centric Water Docking

**Figure 7:**
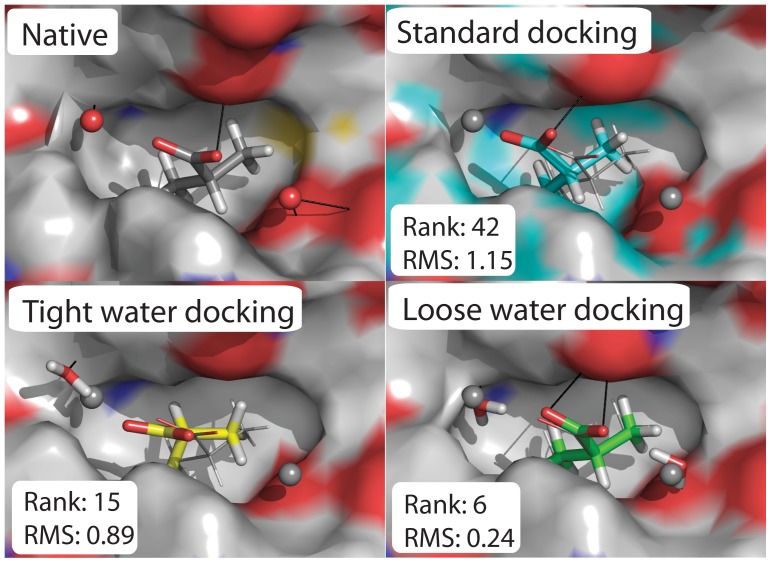
Docking results for CSAR complex ‘set1_120’. Top left: Experimental structure of PDB: 1IUP, coded as CSAR datapoint ‘set1_120’. Waters (Oxygen only) are shown as red spheres. Black lines represent polar contacts predicted by PyMOL. Top right and bottom row: native ligand (lines) and waters (spheres) are shown in grey for comparison. Docked waters are shown as sticks (note that Rosetta adds hydrogens). Docked ligands are shown in cyan, yellow, and green. For each study the models were sorted by total score, then interface energy. The first model with RMSD <2.0 Å is depicted. Its position in the sorted list (rank) and its RMSD to native are shown.

**Figure 8:**
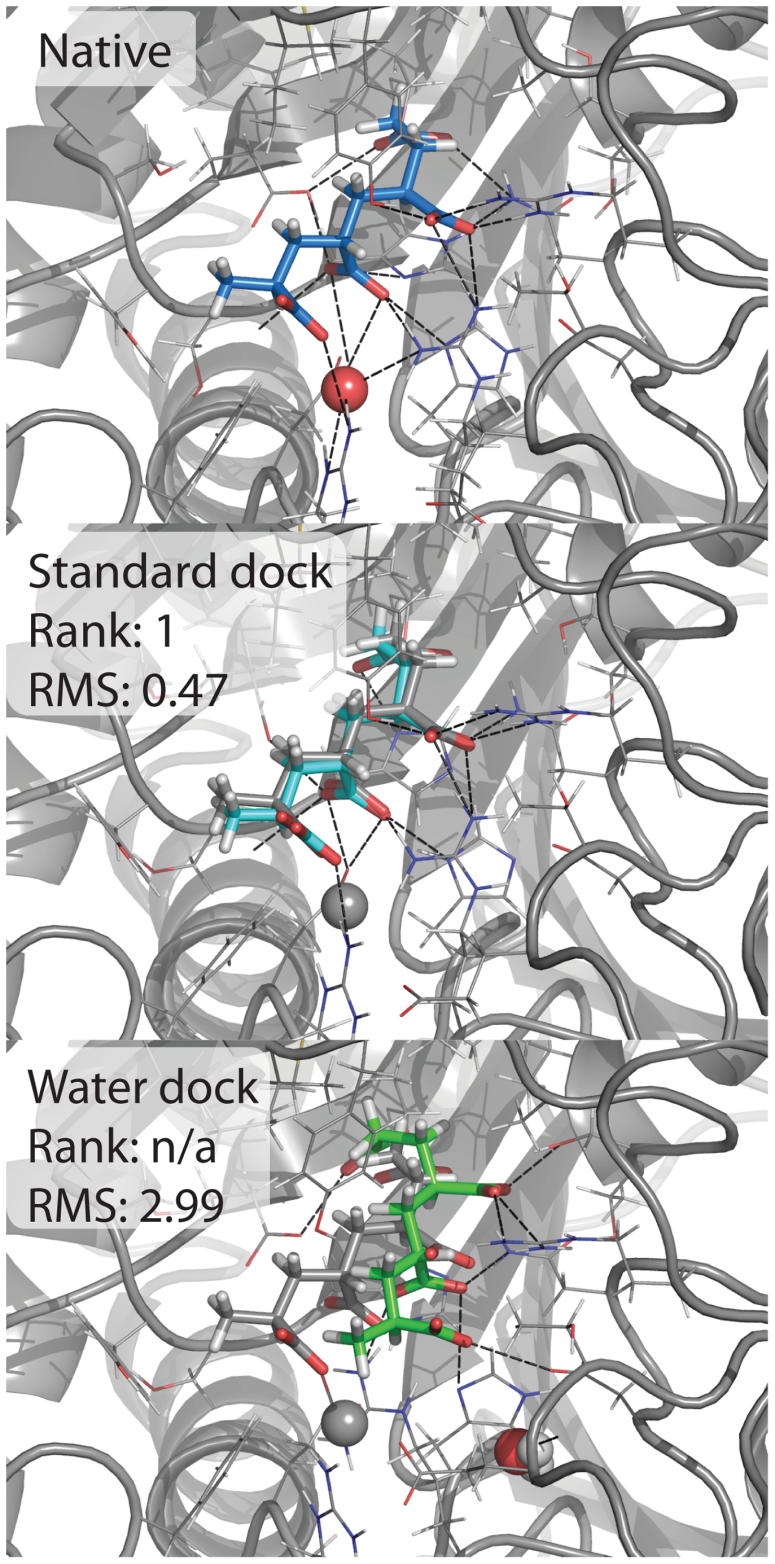
Docking results for CSAR complex set1_181. Top: experimental structure with ligand in blue, water as a red sphere, and polar contacts as black dashed lines. 22 polar contacts are predicted by PyMOL, 4 of which contact the water molecule. Middle: Top scoring model from docking without water. Native ligand and water in grey, Rosetta model in cyan. PyMOL predicts 16 polar contacts. Bottom: Lowest RMSD model from docking with loose waters. Rosetta model shown in green. No model within the top 100 by total energy score has RMSD <2.0 Å (hence rank is ‘n/a’). Shown is the lowest RMSD structure. PyMOL predicts 11 polar contacts (1 with water).

Figure 7 illustrates a case in which ligand-centric waters (which move with the ligand during ligand translation and rotation) restrict sampling of incorrect ligand binding poses and lead to improved ranking. In this case, water reduces the availability of non-clashing poses, thus increasing the likelihood of finding the correct pose. In contrast, Figure 8 reveals a case where standard docking succeeds and docking with the inclusion of water fails. In this case the native structure contains 22 PyMOL [26] predicted polar contacts between ligand and protein, making the binding site very crowded. These are predicted using the PyMOL distance command, mode = 2; h_bond_cutoff_center set to 3.6 Å and h_bond_cutoff_edge set to 3.2 Å. The complexity of the hydrogen bonding network makes side chain rotamer packing especially reliant on the initial positions of the small molecule and water. Using a protocol that produces only 1000 models, Rosetta fails to sample an accurate enough position for both ligand and water necessary to recapitulate the hydrogen bonding network during rotamer packing. In such crowded interfaces, additional low resolution sampling may be necessary to correctly place both water and ligand.

**Figure 9:**
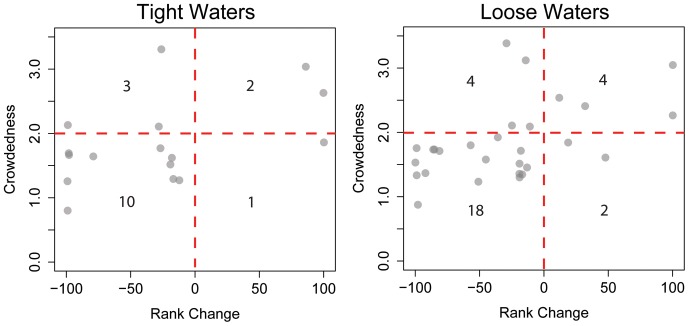
Relation between binding pocket crowdedness and the improvements in CSAR model ranking when water is docked. Crowdedness is calculated as the number of ligand/protein contacts divided by the total number of ligand atoms. Datapoints with rank changes between −10 and 10 were omitted to focus on data where water docking makes a large impact on results. Note that below a crowdedness threshold of 2, addition of water rarely worsens rank.

Change in rank between standard and ligand-centric water docking is plotted in Figure 9. Crowdedness is calculated as the number of pymol predicted ligand/protein polar contacts divided by the total number of ligand atoms. Below a crowdedness threshold of two, ranks improve in 10 out of 11 (tight water) or 18 out of 20 (loose water) cases. Above the threshold rank improvements are as common as worsening of rank. Thus water docking is more likely to improve ranks in spacious binding pockets. However crowded interfaces require a combined accuracy of water and ligand placement that may cause water docking to hinder ligand placement.

Thus water docking presents a trade-off between reducing the available pose sampling space, while at the same time increasing the degrees of freedom in the docking study. Throughout this paper we produce 1000 models per input, regardless of how many water molecules are present and regardless of whether water molecules are initialized at crystallographic or random positions. Scaling the number of models built and whether likely positions are known from crystallography is expected to increase success rates.

### Ligand-centric Docking of Interface Waters does not Affect Binding Affinity Predictions

R values between experimental and predicted binding affinity for the ‘tight’ subset are 0.54 (standard dock) and 0.51 (water dock). For the ‘loose’ subset these values are 0.54 (standard dock) and 0.46 (water dock). Thus while RMSD and rank metrics improve, binding affinity prediction does not. This may be due to the fact that Rosetta score terms weights have already been adjusted to account for the effects of water. For instance hydrogen bond weights have been optimized to account for the change in energy compared to hydrogen bonds formed with water [10]. Similarly the “hydrophobic” score terms are used to represent desolvation of the protein receptor. Further improvements to RosettaLigand water docking will include re-optimizing the Rosetta score function to appropriately evaluate the effects of explicit water on free energy. We suggest that successes in RMSD and rank metrics are gained mainly because of improved sampling, rather than improved scoring. Water most likely plays an indirect role in improving Rosetta scoring (see Table 3), by leading Rosetta to more accurate ligand placement and subsequent selection of side chain conformations.

### Conclusion

In conclusion, where comparative models or experimental data sheds light on the rough position of interface waters relative to a protein or a ligand, including those waters in Rosetta docking studies can significantly improve prediction results. Our findings are especially significant since we include protein side-chain and backbone flexibility as well as ligand flexibility. Additionally we use a much larger dataset of 341 diverse protein/ligand complexes [1] to provide a more stringent and comprehensive benchmark than previous studies. RosettaLigand *protein-centric* water docking is particularly useful where the protein binding site is well characterized (e.g. HIV-1 PR). *Ligand-centric* water docking enables improvements in inhibitor placement by more effectively filling spacious binding pockets, thereby reducing the available pose sampling space.

In the most crowded binding pockets, explicit waters may decrease the probability of successful docking, due to the additional sampling needed to simultaneously identify correct positions for ligand and water. In our study, waters uniformly sampled positions within a 4 A (protein-centric) or 1 A (ligand-centric) radius sphere. A future direction will be to predict water positions without prior knowledge. Future work will also include efforts to determine whether water docking can improve selection of the correct ligand conformer from a library of conformations. Our study was restricted to waters that bridge hydrogen bonding interactions between protein and ligand. Yet networks of water/water hydrogen bonds can contribute to the stability of the complex by keeping bridging water molecules in the right position [27]. Water molecules can also bridge protein/protein interactions, further stabilizing protein conformation [28], [29]. Such complex networks are beyond the scope of this study.

While we demonstrate that water docking can improve inhibitor placement, we did not see a significant improvement in binding affinity prediction. Re-optimizing the Rosetta score function for the inclusion of water molecules may reveal improvement in this area. However, in order to more accurately estimate binding affinities, it will be necessary to consider not only the coordination of waters between the protein and the ligand, but also the release of waters from solvated unbound ligand and unbound protein. This release can lead to an entropy gain of up to 2 kcal/mol per water, but is accompanied by enthalpic costs [30].

In the evolution of protein/ligand interfaces the balance of entropy-enthalpy requirements of ligand binding [7] could be fine-tuned through removal or addition of water. Addition of water molecules increases the chance for a protein to evolve to recognize a specific small molecule as a larger number of favorable protein/ligand/water arrangements is possible than just protein/ligand complexes. In contrast, human-designed interactions of proteins with ligands (drug discovery) might have fewer water molecules in the interface because in structure-based computer-aided drug design waters have typically been ignored [31]. With continued progress in docking with explicit waters we hope to overcome this limitation.

Improvements in binding affinity predictions will need to address the effect of solvation on entropy as well as enthalpy.

## Supporting Information

File S1 A combined supporting information file (File S1) has been prepared. This file includes the following Figures and Tables. Table S1. Change in RMSD of top HIV-1 PR/PI models when water is docked. RMSDs are calculated between inhibitor atoms of top scoring Rosetta model and experimentally determined structure. In each column the number to the left indicates the RMSD of the top scoring Rosetta model using standard docking. The number to the right indicates the change in RMSD seen in the top scoring model when water is added to the docking study (protein-centric water docking). A ‘+’ indicates water docking worsened the result, while a ‘−’ sign indicates an improvement in RMSD upon water docking. In green are studies where adding water improved inhibitor RMSD by greater than 1 Å. In these cross-docking studies, the inhibitor shown in column 2 was docked into the protein structure shown in row 1. **Table S2. RMSDs between HIV-1 protease input PDBs.** Column and row headers correspond to “ID” from table S1. RMSDs are calculated from 3 different atom selections. **Table S3. Probabilities of docking success & failure given various sample sizes.** Success is defined as the RMSD between the experimental inhibitor coordinates and the top scoring Rosetta model being below 2.0 Å. **Table S4. Equations for best-fit lines shown in** **Figure 6** **. Figure S1. CSAR inhibitor properties and ‘loose water’ count.** The width of each bar indicates the number of CSAR datapoints the bar summarizes. Number of interface waters is indicated on the X-axis. The solid black line within the box represents the median. The top and bottom of the box represent the 25^th^ and 75^th^ percentile, the dotted lines extend to the min and max values. Outliers are plotted as black dots and calculated as values less than less than Q1–1.5*IQR or greater than Q3+1.5*IQR. On the Y-axis, various inhibitor properties are shown. **Figure S2. CSAR inhibitor properties and ‘tight water’ count.** See caption to Figure 1. Tight waters differ from loose waters in that they must be within 3.0 Å of at least 2 inhibitor and 2 protein atoms (rather than just 1 of each). **Protocol S1. Standard docking XML. Protocol S2. Protein-centric docking XML. Protocol S3. Ligand-centric docking XML. Protocol S4. File-prep, command-line, and post-processing tips.**
(DOCX)
