# Supplementary material for: Towards Ligand Docking Including Explicit Interface Water Molecules
Source: PLoS One. 2013 Jun 28;8(6):e67536. doi: 10.1371/journal.pone.0067536 (PMC3695863; doi:10.1371/journal.pone.0067536)
Supplement: File S1 — A combined supporting information file (File S1) has been prepared. This file includes the following Figures and Tables. Table S1. Change in RMSD of top HIV-1 PR/PI models when water is docked. RMSDs are calculated between inhibitor atoms of top scoring Rosetta model and experimentally determined structure. In each column the number to the left indicates the RMSD of the top scoring Rosetta model using standard docking. The number to the right indicates the change in RMSD seen in the top scoring model when water is added to the docking study (protein-centric water docking). A ‘+’ indicates water docking worsened the result, while a ‘−’ sign indicates an improvement in RMSD upon water docking. In green are studies where adding water improved inhibitor RMSD by greater than 1 Å. In these cross-docking studies, the inhibitor shown in column 2 was docked into the protein structure shown in row 1. Table S2. RMSDs between HIV-1 protease input PDBs. Column and row headers correspond to “ID” from table S1. RMSDs are calculated from 3 different atom selections. Table S3. Probabilities of docking success & failure given various sample sizes. Success is defined as the RMSD between the experimental inhibitor coordinates and the top scoring Rosetta model being below 2.0 Å. Table S4. Equations for best-fit lines shown in Figure 6 . Figure S1. CSAR inhibitor properties and ‘loose water’ count. The width of each bar indicates the number of CSAR datapoints the bar summarizes. Number of interface waters is indicated on the X-axis. The solid black line within the box represents the median. The top and bottom of the box represent the 25th and 75th percentile, the dotted lines extend to the min and max values. Outliers are plotted as black dots and calculated as values less than less than Q1–1.5*IQR or greater than Q3+1.5*IQR. On the Y-axis, various inhibitor properties are shown. Figure S2. CSAR inhibitor properties and ‘tight water’ count. See caption to Figure 1. Tight waters differ fr [file pone.0067536.s001.docx]

**Supplemental Information**

**Contents**

1. Table S1. Change in RMSD of top HIV-1 PR/PI models when water is docked.
2. Table S2. RMSDs between HIV-1 protease input PDBs.
3. Table S3. Probabilities of docking success & failure given various sample sizes.
4. Table S4. Equations for best-fit lines shown in Figure 6.
5. Figure S1. CSAR inhibitor properties and ‘loose water’ count.
6. Figure S2. CSAR inhibitor properties and ‘loose water’ count.
7. Protocol S1. Standard docking XML
8. Protocol S2. Protein-centric docking XML
9. Protocol S3. Ligand-centric docking XML
10. Protocol S4. File-prep, command-line, and post-processing tips

| Table S1. Change in RMSD of top HIV-1 PR/PI models when water is docked. RMSDs are calculated between inhibitor atoms of top scoring Rosetta model and experimentally determined structure. In each column the number to the left indicates the RMSD of the top scoring Rosetta model using standard docking. The number to the right indicates the change in RMSD seen in the top scoring model when water is added to the docking study (protein-centric water docking). A ‘+’ indicates water docking worsened the result, while a ‘-’ sign indicates an improvement in RMSD upon water docking. In green are studies where adding water improved inhibitor RMSD by greater than 1 Å. In these cross-docking studies, the inhibitor shown in column 2 was docked into the protein structure shown in row 1. | | | | | | | | | | |
| --- | --- | --- | --- | --- | --- | --- | --- | --- | --- | --- |
| ID | Ligand/Protein | 1HXW | 1KZK | 1LZQ | 1OHR | 1SDT | 1T7J | 2NMW | 2O4S | 5HVP |
| 1 | 1HXW-Ritona | 0.94-0.11 | 2.88-0.95 | 2.54-0.9 | 0.48+0.3 | 0.83-0.1 | 1.83-0.19 | 1.76+0.26 | 2.23-1.27 | 0.65-0.26 |
| 2 | 1KZK-AG1776 | 0.76+0.63 | 2.6-0.09 | 1.97-0.56 | 1.15-0.59 | 0.75-0.11 | 1.86-0.87 | 1.15-0.18 | 1.37-0.45 | 0.71-0.34 |
| 3 | 1KZK-KNI272 | 1.01+0.47 | 10.5-8.26 | 1.54-0.19 | 1.16-0.57 | 0.85+0.14 | 1.77-0.72 | 1.02-0.41 | 2.1-1.07 | 0.58-0.13 |
| 4 | 1KZK-KNI764 | 0.93+0.69 | 1.57+0.71 | 1.7-0.27 | 0.95-0.54 | 1-0.68 | 1.84-0.44 | 1.34+0.1 | 1.53-0.42 | 0.46+0.05 |
| 5 | 1LZQ-Ethyle | 0.81+0.02 | 4.52-2.77 | 1.69-0.04 | 0.84-0.05 | 0.81-0.19 | 2.77-1.55 | 2.65-0.59 | 1.76+0.16 | 0.5+0.16 |
| 6 | 1OHR-Nelfin | 1.01-0.62 | 1.97+0.73 | 0.89-0.21 | 0.74-0.42 | 0.76+0.29 | 2.25-1.14 | 2.05-1.34 | 1.3-0.01 | 0.51+0.03 |
| 7 | 1SDT-Indina | 0.81-0.18 | 2.66-0.96 | 1.73-0.16 | 1.13-0.23 | 0.68-0.25 | 1.63-0.63 | 2.83-0.51 | 2.09-0.37 | 0.65-0.32 |
| 8 | 1T7J-Ampren | 0.84-0.03 | 2.58-0.09 | 0.88+0.99 | 0.61+0.45 | 0.98-0.12 | 1.49-0.46 | 1.47+0 | 2.5-1.06 | 0.69+0.25 |
| 9 | 2NMW-Saquin | 0.82-0.3 | 2.12+0.82 | 2.42+0.26 | 1.42+0.91 | 1.54+0.51 | 1.4-0.19 | 2.4+0.26 | 1.61+1.08 | 0.57-0.02 |
| 10 | 2O4S-Lopina | 0.87-0.21 | 3.02-0.96 | 3.52-1.01 | 0.76-0.17 | 0.82-0.34 | 1.18-0.05 | 2.06-0.52 | 2.71-0.42 | 0.51+0.15 |
| 11 | 5HVP-Acetyl | 1.07+0.01 | 3.11-0.25 | 2.29+0.73 | 1.2-0.03 | 0.89+0.07 | 2.41-0.27 | 2.97+0.42 | 2.03+0.46 | 0.78-0.04 |

| **Table S2. RMSDs between HIV-1 protease input PDBs.** Column and row headers correspond to “ID” from table S1. RMSDs are calculated from 3 different atom selections. | | | | | | | | | | | | | | |
| --- | --- | --- | --- | --- | --- | --- | --- | --- | --- | --- | --- | --- | --- | --- |
| **All C_alpha atoms** | | | | | | | | | | | | | | |
|  | 1 | | 2 | | 3 | | 4 | 5 | 6 | 7 | 8 | 9 | 10 | 11 |
| 1 | 0.00 | |  | |  | |  |  |  |  |  |  |  |  |
| 2 | 0.67 | | 0.00 | |  | |  |  |  |  |  |  |  |  |
| 3 | 0.67 | | 0.00 | | 0.00 | |  |  |  |  |  |  |  |  |
| 4 | 0.67 | | 0.00 | | 0.00 | | 0.00 |  |  |  |  |  |  |  |
| 5 | 0.55 | | 0.51 | | 0.51 | | 0.51 | 0.00 |  |  |  |  |  |  |
| 6 | 0.63 | | 0.61 | | 0.61 | | 0.61 | 0.62 | 0.00 |  |  |  |  |  |
| 7 | 0.46 | | 0.68 | | 0.68 | | 0.68 | 0.59 | 0.77 | 0.00 |  |  |  |  |
| 8 | 0.52 | | 0.49 | | 0.49 | | 0.49 | 0.49 | 0.55 | 0.69 | 0.00 |  |  |  |
| 9 | 0.75 | | 0.63 | | 0.63 | | 0.63 | 0.49 | 0.73 | 0.71 | 0.71 | 0.00 |  |  |
| 10 | 0.36 | | 0.70 | | 0.70 | | 0.70 | 0.60 | 0.79 | 0.34 | 0.65 | 0.77 | 0.00 |  |
| 11 | 0.49 | | 0.69 | | 0.69 | | 0.69 | 0.59 | 0.73 | 0.53 | 0.63 | 0.76 | 0.47 | 0.00 |
|  |  | |  | |  | |  |  |  |  |  |  |  |  |
| **All backbone atoms** | | | | | | | | | | | | | | |
|  | 1 | 2 | | 3 | | | 4 | 5 | 6 | 7 | 8 | 9 | 10 | 11 |
| 1 | 0.00 |  | |  | | |  |  |  |  |  |  |  |  |
| 2 | 0.65 | 0.00 | |  | | |  |  |  |  |  |  |  |  |
| 3 | 0.65 | 0.00 | | 0.00 | | |  |  |  |  |  |  |  |  |
| 4 | 0.65 | 0.00 | | 0.00 | | | 0.00 |  |  |  |  |  |  |  |
| 5 | 0.54 | 0.50 | | 0.50 | | | 0.50 | 0.00 |  |  |  |  |  |  |
| 6 | 0.62 | 0.59 | | 0.59 | | | 0.59 | 0.61 | 0.00 |  |  |  |  |  |
| 7 | 0.45 | 0.66 | | 0.66 | | | 0.66 | 0.58 | 0.76 | 0.00 |  |  |  |  |
| 8 | 0.50 | 0.48 | | 0.48 | | | 0.48 | 0.48 | 0.54 | 0.67 | 0.00 |  |  |  |
| 9 | 0.74 | 0.62 | | 0.62 | | | 0.62 | 0.49 | 0.72 | 0.70 | 0.70 | 0.00 |  |  |
| 10 | 0.35 | 0.68 | | 0.68 | | | 0.68 | 0.58 | 0.77 | 0.33 | 0.63 | 0.75 | 0.00 |  |
| 11 | 0.48 | 0.68 | | 0.68 | | | 0.68 | 0.58 | 0.72 | 0.51 | 0.62 | 0.74 | 0.46 | 0.00 |
|  |  |  | |  | | |  |  |  |  |  |  |  |  |
| **Non-hydrogen atoms of residues within 6 Å of inhibitor (in the case of sequence identity mismatch, side-chain atoms are not included in the RMSD calculation)** | | | | | | | | | | | | | | |
|  | 1 | 2 | | 3 | | 4 | | 5 | 6 | 7 | 8 | 9 | 10 | 11 |
| 1 | 0.00 |  | |  | |  | |  |  |  |  |  |  |  |
| 2 | 0.79 | 0.00 | |  | |  | |  |  |  |  |  |  |  |
| 3 | 0.79 | 0.00 | | 0.00 | |  | |  |  |  |  |  |  |  |
| 4 | 0.79 | 0.00 | | 0.00 | | 0.00 | |  |  |  |  |  |  |  |
| 5 | 0.78 | 0.80 | | 0.80 | | 0.80 | | 0.00 |  |  |  |  |  |  |
| 6 | 0.61 | 0.89 | | 0.89 | | 0.89 | | 0.93 | 0.00 |  |  |  |  |  |
| 7 | 0.90 | 0.71 | | 0.71 | | 0.71 | | 0.89 | 0.94 | 0.00 |  |  |  |  |
| 8 | 0.91 | 0.70 | | 0.70 | | 0.70 | | 0.80 | 1.02 | 0.86 | 0.00 |  |  |  |
| 9 | 0.78 | 0.94 | | 0.94 | | 0.94 | | 0.83 | 0.75 | 0.87 | 1.02 | 0.00 |  |  |
| 10 | 0.63 | 0.75 | | 0.75 | | 0.75 | | 0.94 | 0.90 | 0.79 | 1.00 | 0.89 | 0.00 |  |
| 11 | 0.90 | 1.05 | | 1.05 | | 1.05 | | 1.00 | 0.98 | 1.05 | 1.09 | 0.92 | 0.92 | 0.00 |

**Table S3. Probabilities of docking success & failure given various sample sizes.** Success is defined as the RMSD between the experimental inhibitor coordinates and the top scoring Rosetta model being below 2.0 Å.

| N | Success | Failure | SS | FF | S∩F | F∩S | S\|S | F\|F | S\|F | F\|S |
| --- | --- | --- | --- | --- | --- | --- | --- | --- | --- | --- |
| 1 | 0.072 | 0.928 | 0.009 | 0.865 | 0.063 | 0.063 | 0.125 | 0.932 | 0.068 | 0.875 |
| 2 | 0.128 | 0.872 | 0.027 | 0.771 | 0.101 | 0.101 | 0.211 | 0.884 | 0.116 | 0.789 |
| 5 | 0.250 | 0.750 | 0.094 | 0.595 | 0.156 | 0.156 | 0.376 | 0.793 | 0.208 | 0.624 |
| 10 | 0.379 | 0.621 | 0.199 | 0.441 | 0.180 | 0.180 | 0.525 | 0.710 | 0.290 | 0.475 |
| 25 | 0.568 | 0.432 | 0.407 | 0.271 | 0.161 | 0.161 | 0.717 | 0.627 | 0.373 | 0.283 |
| 50 | 0.686 | 0.314 | 0.568 | 0.196 | 0.118 | 0.118 | 0.828 | 0.624 | 0.376 | 0.172 |
| 100 | 0.762 | 0.238 | 0.688 | 0.164 | 0.074 | 0.074 | 0.903 | 0.689 | 0.311 | 0.097 |
| 200 | 0.795 | 0.205 | 0.748 | 0.157 | 0.048 | 0.048 | 0.941 | 0.766 | 0.232 | 0.060 |
| 300 | 0.812 | 0.188 | 0.779 | 0.154 | 0.033 | 0.033 | 0.959 | 0.819 | 0.176 | 0.041 |
| 400 | 0.817 | 0.183 | 0.794 | 0.161 | 0.023 | 0.023 | 0.972 | 0.880 | 0.123 | 0.028 |

**Table S4. Equations for best-fit lines shown in Figure 6.** These values were generated using tools available through ZunZun.com.

| Best-fit line | P(S\|F) | P(F\|S) |
| --- | --- | --- |
| Equation | 1.0/(a+((x-b)/c)^d) + offset | a*exp(-0.5*((ln(x-d)-b)/c)^2)+offset |
| a | 0.450 | 1.137 |
| b | 3.632 | 0.961 |
| c | 1.853 | 9.283 |
| d | -1.218 | 0.925 |
| offset | -0.071 | 0.001 |

**Figure S1. CSAR inhibitor properties and ‘loose water’ count.** The width of each bar indicates the number of CSAR datapoints the bar summarizes. Number of interface waters is indicated on the X-axis. The solid black line within the box represents the median. The top and bottom of the box represent the 25^th^ and 75^th^ percentile, the dotted lines extend to the min and max values. Outliers are plotted as black dots and calculated as values less than less than Q1 - 1.5*IQR or greater than Q3 + 1.5*IQR. On the Y-axis, various inhibitor properties are shown.


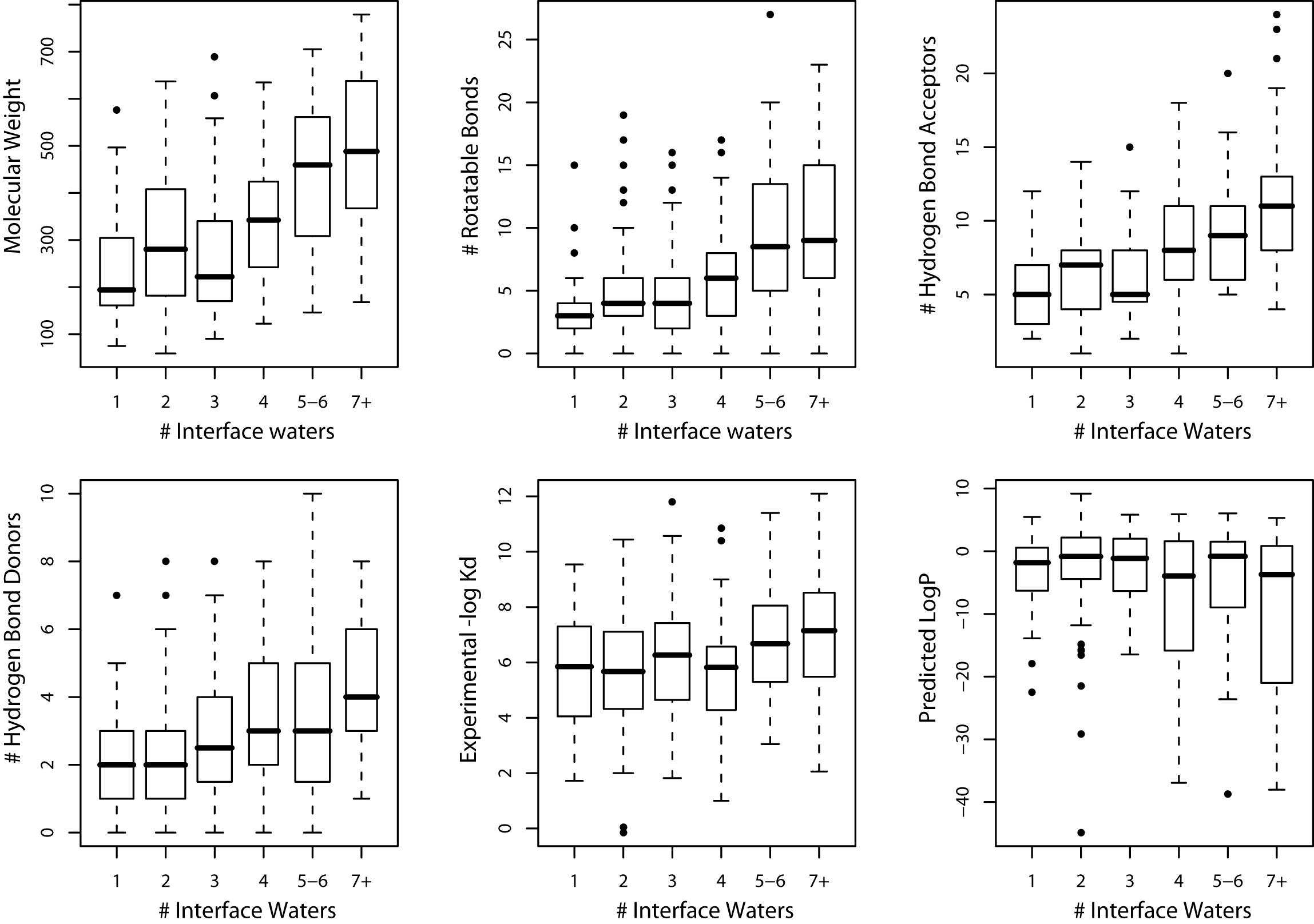


**Figure S2.** **CSAR inhibitor properties and ‘tight water’ count.** See caption to Figure 1. Tight waters differ from loose waters in that they must be within 3.0 Å of at least 2 inhibitor and 2 protein atoms (rather than just 1 of each).


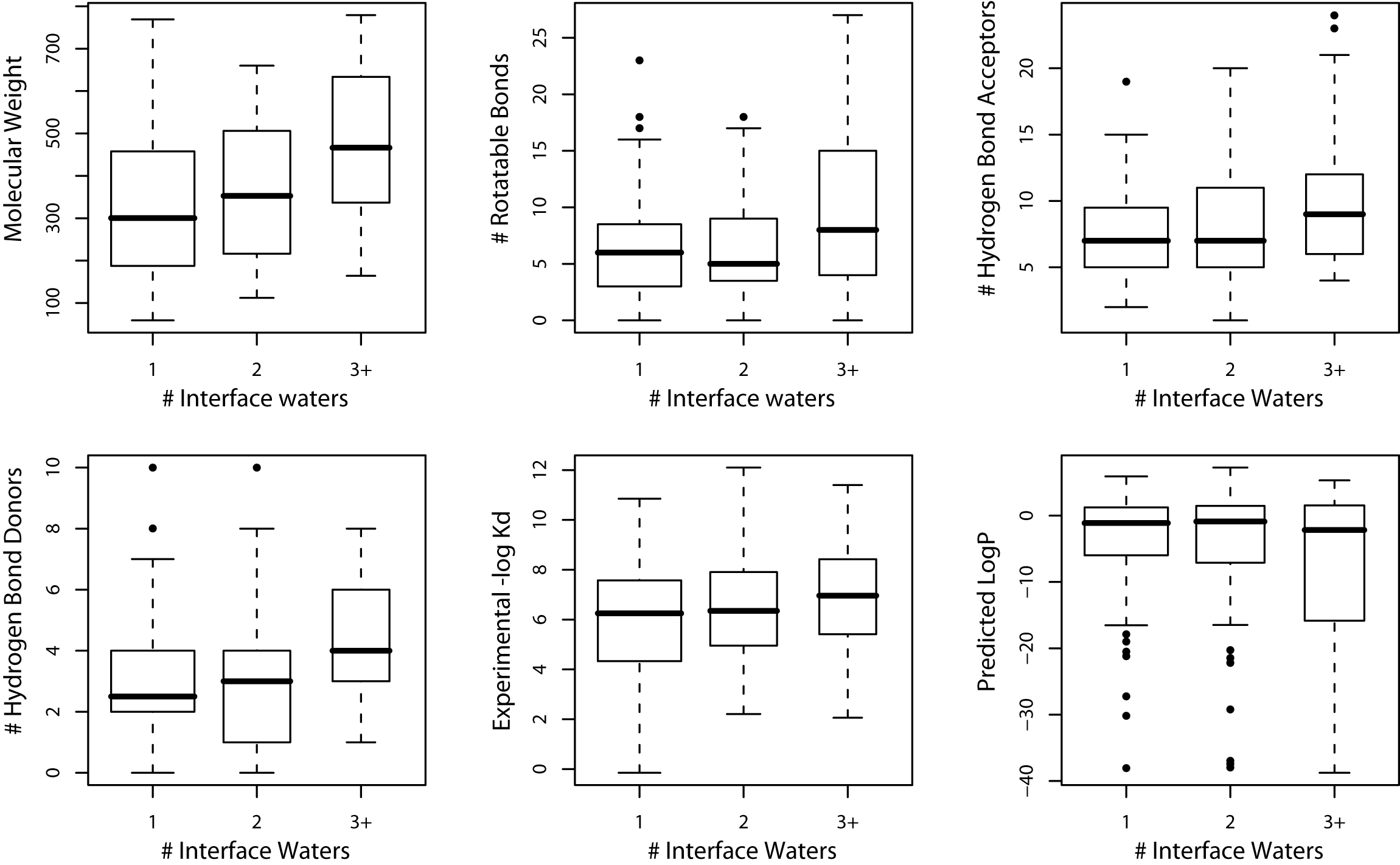


**Protocol S1: Standard docking XML**

<ROSETTASCRIPTS>
 <SCOREFXNS>
 <ligand_soft_rep weights=ligand_soft_rep>
 <Reweight scoretype=hack_elec weight=0.42/>
 <Reweight scoretype=hbond_bb_sc weight=1.3/>
 <Reweight scoretype=hbond_sc weight=1.3/>
 <Reweight scoretype=rama weight=0.2/>
 </ligand_soft_rep>
 <hard_rep weights=ligand>
 <Reweight scoretype=fa_intra_rep weight=0.004/>
 <Reweight scoretype=hack_elec weight=0.42/>
 <Reweight scoretype=hbond_bb_sc weight=1.3/>
 <Reweight scoretype=hbond_sc weight=1.3/>
 <Reweight scoretype=rama weight=0.2/>
 </hard_rep>
 </SCOREFXNS>
 <LIGAND_AREAS>
 <inhibitor_dock_sc chain=X cutoff=6.0 add_nbr_radius=true all_atom_mode=true/>
 <inhibitor_final_sc chain=X cutoff=6.0 add_nbr_radius=true all_atom_mode=true/>
 <inhibitor_final_bb chain=X cutoff=7.0 add_nbr_radius=false all_atom_mode=true Calpha_restraints=0.3/>
 </LIGAND_AREAS>
 <INTERFACE_BUILDERS>
 <side_chain_for_docking ligand_areas=inhibitor_dock_sc/>
 <side_chain_for_final ligand_areas=inhibitor_final_sc/>
 <backbone ligand_areas=inhibitor_final_bb extension_window=3/>
 </INTERFACE_BUILDERS>
 <MOVEMAP_BUILDERS>
 <docking sc_interface=side_chain_for_docking minimize_water=false/>
 <final sc_interface=side_chain_for_final bb_interface=backbone minimize_water=false/>
 </MOVEMAP_BUILDERS>
 <MOVERS>
 <Translate name=translate chain=X distribution=uniform angstroms=5 cycles=50 force=true/> first place the ligand
 <Rotate name=rotate chain=X distribution=uniform degrees=360 cycles=800/>
 <SlideTogether name=slide_together chains=X/>
 <HighResDocker name=high_res_docker cycles=6 repack_every_Nth=3 scorefxn=ligand_soft_rep movemap_builder=docking/>
 <FinalMinimizer name=final scorefxn=hard_rep movemap_builder=final/>
 <InterfaceScoreCalculator name=add_scores chains=X scorefxn=hard_rep/>
 </MOVERS>
 <PROTOCOLS>
 <Add mover_name=translate/>
 <Add mover_name=rotate/>
 <Add mover_name=slide_together/>
 <Add mover_name=high_res_docker/>
 <Add mover_name=final/>
 <Add mover_name=add_scores/>
 </PROTOCOLS>
</ROSETTASCRIPTS>

**Protocol S2: Protein-centric docking XML**

<ROSETTASCRIPTS>
 <SCOREFXNS>
 <ligand_soft_rep weights=ligand_soft_rep>
 <Reweight scoretype=hack_elec weight=0.42/>
 <Reweight scoretype=hbond_bb_sc weight=1.3/>
 <Reweight scoretype=hbond_sc weight=1.3/>
 <Reweight scoretype=rama weight=0.2/>
 </ligand_soft_rep>
 <hard_rep weights=ligand>
 <Reweight scoretype=fa_intra_rep weight=0.004/>
 <Reweight scoretype=hack_elec weight=0.42/>
 <Reweight scoretype=hbond_bb_sc weight=1.3/>
 <Reweight scoretype=hbond_sc weight=1.3/>
 <Reweight scoretype=rama weight=0.2/>
 </hard_rep>
 </SCOREFXNS>
 <LIGAND_AREAS>
 <inhibitor_dock_sc chain=X cutoff=6.0 add_nbr_radius=true all_atom_mode=true/>
 <water_dock_sc chain=W cutoff=2.0 add_nbr_radius=true all_atom_mode=true/>
 <inhibitor_final_sc chain=X cutoff=6.0 add_nbr_radius=true all_atom_mode=true/>
 <water_final_sc chain=W cutoff=2.0 add_nbr_radius=true all_atom_mode=true/>
 <inhibitor_final_bb chain=X cutoff=7.0 add_nbr_radius=false all_atom_mode=true Calpha_restraints=0.3/>
 <water_final_bb chain=W cutoff=2.5 add_nbr_radius=false all_atom_mode=true Calpha_restraints=0.3/>
 </LIGAND_AREAS>
 <INTERFACE_BUILDERS>
 <side_chain_for_docking ligand_areas=inhibitor_dock_sc,water_dock_sc/>
 <side_chain_for_final ligand_areas=inhibitor_final_sc,water_final_sc/>
 <backbone ligand_areas=inhibitor_final_bb,water_final_bb extension_window=3/>
 </INTERFACE_BUILDERS>
 <MOVEMAP_BUILDERS>
 <docking sc_interface=side_chain_for_docking minimize_water=true/>
 <final sc_interface=side_chain_for_final bb_interface=backbone minimize_water=true/>
 </MOVEMAP_BUILDERS>
 <MOVERS>
 <Translate name=translate_x chain=X distribution=uniform angstroms=5.0 cycles=50/>
 <CompoundTranslate name=compound_translate randomize_order=true allow_overlap=false>
 <Translates chains=W distribution=uniform angstroms=4.0 cycles=50/>
 </CompoundTranslate>
 <Rotate name=rotate_x chain=X distribution=uniform degrees=360 cycles=800/>
 <Rotate name=rotate_w chain=W distribution=uniform degrees=360 cycles=100/>
 <SlideTogether name=slide_together chains=X,W/>
 <HighResDocker name=high_res_docker cycles=6 repack_every_Nth=3 scorefxn=ligand_soft_rep movemap_builder=docking/>
 <FinalMinimizer name=final scorefxn=hard_rep movemap_builder=final/>
 <InterfaceScoreCalculator name=add_scores chains=X,W scorefxn=hard_rep/>
 </MOVERS>
 <PROTOCOLS>
 <Add mover_name=translate_x/>
 <Add mover_name=rotate_x/>
 <Add mover_name=translate_w/>
 <Add mover_name=rotate_w/>
 <Add mover_name=slide_together/>
 <Add mover_name=high_res_docker/>
 <Add mover_name=final/>
 <Add mover_name=add_scores/>
 <Add mover_name=report_scores/>
 </PROTOCOLS>
</ROSETTASCRIPTS>

**Protocol S3: Ligand-centric XML**

<ROSETTASCRIPTS>
 <SCOREFXNS>
 <ligand_soft_rep weights=ligand_soft_rep>
 <Reweight scoretype=hack_elec weight=0.42/>
 <Reweight scoretype=hbond_bb_sc weight=1.3/>
 <Reweight scoretype=hbond_sc weight=1.3/>
 <Reweight scoretype=rama weight=0.2/>
 </ligand_soft_rep>
 <hard_rep weights=ligand>
 <Reweight scoretype=fa_intra_rep weight=0.004/>
 <Reweight scoretype=hack_elec weight=0.42/>
 <Reweight scoretype=hbond_bb_sc weight=1.3/>
 <Reweight scoretype=hbond_sc weight=1.3/>
 <Reweight scoretype=rama weight=0.2/>
 </hard_rep>
 </SCOREFXNS>
 <LIGAND_AREAS>
 <inhibitor_dock_sc chain=X cutoff=6.0 add_nbr_radius=true all_atom_mode=true/>
 <water_dock_sc chain=W cutoff=2.0 add_nbr_radius=true all_atom_mode=true/>
 <inhibitor_final_sc chain=X cutoff=6.0 add_nbr_radius=true all_atom_mode=true/>
 <water_final_sc chain=W cutoff=2.0 add_nbr_radius=true all_atom_mode=true/>
 <inhibitor_final_bb chain=X cutoff=7.0 add_nbr_radius=false all_atom_mode=true Calpha_restraints=0.3/>
 <water_final_bb chain=W cutoff=2.5 add_nbr_radius=false all_atom_mode=true Calpha_restraints=0.3/>
 </LIGAND_AREAS>
 <INTERFACE_BUILDERS>
 <side_chain_for_docking ligand_areas=inhibitor_dock_sc,water_dock_sc/>
 <side_chain_for_final ligand_areas=inhibitor_final_sc,water_final_sc/>
 <backbone ligand_areas=inhibitor_final_bb,water_final_bb extension_window=3/>
 </INTERFACE_BUILDERS>
 <MOVEMAP_BUILDERS>
 <docking sc_interface=side_chain_for_docking minimize_water=true/>
 <final sc_interface=side_chain_for_final bb_interface=backbone minimize_water=true/>
 </MOVEMAP_BUILDERS>
 <MOVERS>
 <Translate name=translate_X chain=W distribution=uniform angstroms=5 cycles=50 force=true tag_along_chains=W/>
 <CompoundTranslate name=compound_translate randomize_order=true allow_overlap=false>
 <Translates chain=W distribution=uniform angstroms=1 cycles=50 force=true/>
 </CompoundTranslate>
 <Rotate name=rotate_x chain=X distribution=uniform degrees=360 cycles=800/>
 <Rotates name=rotate_w chain=W distribution=uniform degrees=360 cycles=100/>
 <SlideTogether name=slide_together chains=X,W/>
 <HighResDocker name=high_res_docker cycles=6 repack_every_Nth=3 scorefxn=ligand_soft_rep movemap_builder=docking/>
 <FinalMinimizer name=final scorefxn=hard_rep movemap_builder=final/>
 <InterfaceScoreCalculator name=add_scores chains=X,W scorefxn=hard_rep/>
 </MOVERS>
 <PROTOCOLS>
 <Add mover_name=translate_X/>
 <Add mover_name=rotate_x/>
 <Add mover_name=compound_translate/>
 <Add mover_name=rotate_w/>
 <Add mover_name=slide_together/>
 <Add mover_name=high_res_docker/>
 <Add mover_name=final/>
 <Add mover_name=add_scores/>
 </PROTOCOLS>
</ROSETTASCRIPTS>

**Protocol S4. File-prep, command-line, and post-processing tips**

Prepare input PDBs by removing all lines except those that start with ATOM or HETATM. Retain only HETATM lines you wish to include in your analysis. We use pymol to identify waters within 3 angstroms of both the protein and the ligand. A demo directory containing a working example of standard, protein-centric, and ligand-centric docking has been committed to rosetta_demos/protocol_capture/2012/ligand_water_docking. The pymol script used to identify interface waters is provided within the demo directory.

The BCL (www.meilerlab.org) was used to calculate chemical properties reported for the CSAR dataset. The command line we used is reported here:

/sb/meiler/Linux2/x86_64/bcl/current/bin/bcl.exe EditEnsemble -input_filenames <name of mol file> -output_matched test.sdf -add_properties NRotBond HbondAcceptor HbondDonor LogP NAtoms Weight >& /dev/null

Each ligand must be converted to .mol, or .mdl format. These are provided to the script molfile_to_params.py to create Rosetta .params input files and ligand PDBs with Rosetta atom names. The script is available with the Rosetta release in the folder rosetta_source/src/python/apps/public/molfile_to_params.py
